# Estimands in epigenome-wide association studies

**DOI:** 10.1186/s13148-021-01083-9

**Published:** 2021-04-29

**Authors:** Jochen Kruppa, Miriam Sieg, Gesa Richter, Anne Pohrt

**Affiliations:** 1 Charité - University Medicine, Corporate Member of Freie Universität Berlin, Humboldt-Universität zu Berlin, and Berlin Institute of Health, Institute of Biometry and Clinical Epidemiology, Charitéplatz 1, 10117 Berlin, Germany; 2 Berlin Institute of Health (BIH), Anna-Louisa-Karsch-Straße 2, 10178 Berlin, Germany; 3 Department of Periodontology and Synoptic Dentistry, Institute of Dental, Oral and Maxillary Medicine, Charité - University Medicine, Charitéplatz 1, 10117 Berlin, Germany

**Keywords:** Multiple testing, DNA methylation, Reproducible research, Epigenome-wide association study (EWAS), Estimands

## Abstract

**Background:**

In DNA methylation analyses like epigenome-wide association studies, effects in differentially methylated CpG sites are assessed. Two kinds of outcomes can be used for statistical analysis: Beta-values and *M*-values. *M*-values follow a normal distribution and help to detect differentially methylated CpG sites. As biological effect measures, differences of *M*-values are more or less meaningless. Beta-values are of more interest since they can be interpreted directly as differences in percentage of DNA methylation at a given CpG site, but they have poor statistical properties. Different frameworks are proposed for reporting estimands in DNA methylation analysis, relying on Beta-values, *M*-values, or both.

**Results:**

We present and discuss four possible approaches of achieving estimands in DNA methylation analysis. In addition, we present the usage of *M*-values or Beta-values in the context of bioinformatical pipelines, which often demand a predefined outcome. We show the dependencies between the differences in *M*-values to differences in Beta-values in two data simulations: a analysis with and without confounder effect. Without present confounder effects, *M*-values can be used for the statistical analysis and Beta-values statistics for the reporting. If confounder effects exist, we demonstrate the deviations and correct the effects by the intercept method. Finally, we demonstrate the theoretical problem on two large human genome-wide DNA methylation datasets to verify the results.

**Conclusions:**

The usage of *M*-values in the analysis of DNA methylation data will produce effect estimates, which cannot be biologically interpreted. The parallel usage of Beta-value statistics ignores possible confounder effects and can therefore not be recommended. Hence, if the differences in Beta-values are the focus of the study, the intercept method is recommendable. Hyper- or hypomethylated CpG sites must then be carefully evaluated. If an exploratory analysis of possible CpG sites is the aim of the study, *M*-values can be used for inference.

**Supplementary Information:**

The online version contains supplementary material available at 10.1186/s13148-021-01083-9.

## Background

The reporting of estimands, i.e., effect estimates, in DNA methylation analysis is a challenge for scientists. In DNA methylation analysis with DNA microarray data, the scientist can decide between two kinds of reported outcomes of the statistical analysis: differences in Beta-values and differences in *M*-values [1]. Raw data come as methylated and unmethylated intensities per sample. The fraction of methylated to unmethylated probes for a given CpG site is defined by the Beta-values by describing the percentage of DNA methylation for a given CpG site across all DNA molecules in the sample. While Beta-values describe the frequency of DNA methylation at a given CpG site; the *M*-values are standardized Beta-values. The standardization corresponds to a “logit” transformation. Hence, Beta-values follow a beta distribution with the limits at 0 and 1, while *M*-values are theoretically normal distributed real values. Which of the two outcomes, Beta-values or *M*-values, should be analyzed with which method is controversial among bioinformaticians. However, the discussion is somehow hidden in the different bioinformatical analysis pipelines. Here, we want to openly discuss the limitations and inconsistencies. Beta- and *M*-values are often associated with illumina microarray data; however, percentage of methylation and the corresponding “logit”-transformation can also be generated from bisulfite sequencing data.

We assume that the reader is familiar with clinical epigenetics and its potential as a biomarker and importance in heredity. If not, Berdasco and Esteller [2] demonstrate the importance of clinical epigenetics in translation, and Herrel et al. [3] provide a broader perspective of epigenetics in ecology and evolution. Discussing the differences between bisulfite sequencing and DNA methylation microarrays is beyond the scope of this work. We refer to Heiss et al. [4] to track this “battle of epigenetic proportions”.

DNA methylation analysis often focuses on the generation of *p* value sorted lists of CpG sites. Often these lists are adjusted for multiplicity to prevent an inflation of the type I error. These lists have their purpose in downstream pathway analysis. In contrast, Betensky [5] and Wasserstein et al. [6] state that *p* values cannot be interpreted in isolation and must be seen in the context of the design and application including meaningful effect measures. In this work, therefore, we aim to shed light on how meaningful effect estimates for DNA methylation analysis can be achieved. If the research question is focused on *p* value sorted lists of CpG sites, we recommend Van Rooij et al. [7] as a complement to our work.

The proper choice of estimands, i.e., effect estimates, is embedded into a more general discussion on reproducibility. So far, the focus of the estimand discussion is driven by drug development and clinical trials [8]. Akacha et al. [9] state that specific choices in the statistical analysis may blur the scientific question in parts or completely. Hence, there is a need for estimands that properly answer the scientific question. However, the choice of the right estimand in DNA methylation analysis is disputable. We can see the statistical method of estimation as “estimator” and the target of the estimation as “estimand”. The interest reader might consider Mallinckrodt et al. [10] for a deep discussion of estimands, estimators and sensitivity analysis in clinical trials.

Leuchs et al. [11] provide a process chart for the decision of a valid estimand in a clinical trial considering the primary endpoint, the clinical trial design, and the method of analysis. Therefore, it is paramount to discuss the choice of the estimand carefully. The authors do not discuss the topic in the context of genetics, but their considerations are applicable here as well.

In general, any genetic analysis is done in a pipeline-like fashion. This is also true for the analysis of DNA methylation data. Different statistical methods are run in a sequential pattern. For the detection of differentially methylated CpG sites, *M*-values are predominantly used due to their asymptotically normal distributed values and therefore better statistical properties. This is a theoretical statistical argument, which is valid; see Du et al. [1] for a more comprehensive explanation. The analysis of *M*-values and the resulting *p* values is not problematic. But *p* values should be reported together with effect estimates so that clinical relevance can be assessed. The coefficients from the differential analysis are differences in *M*-values. Unfortunately, these differences are not possible to interpret biologically. Thus, if effect estimates are needed, differences in Beta-values—as difference of DNA methylation frequency—could be more sensible as effect measures.

Among others, Du et al. [1] and Maksimovic et al. [12] recommend to use *M*-values for the analysis of differential DNA methylation and Beta-values statistics for reporting to investigators. At first glance, this advice seems reasonable, as it yields significance lists combined with interpretable differences in DNA methylation percentage. But this is only the case, if no confounding is present. Often the analysis on *M*-values is adjusted for batch effects and confounders. However, the raw Beta-values statistics are not adjusted for these effects. Running the analysis on *M*-values and reporting changes as differences in Beta-values implicitly assumes that the data include no confounder effects.

In the past, different approaches were applied in order to circumvent the problem of biologically non-informative effect measures. A beta regression can be calculated on the Beta-values without transforming them to *M*-values [13]. Beta regression delivers directly interpretable effect estimates. This method, however, has severe heteroscedasticity for highly unmethylated or methylated (hypo- and hypermethylated) CpG sites [1]. This method has been applied in different studies [14, 15], with different link functions [16] or with the reporting of both linear and beta regression coefficients [15]. A comprehensive overview and introduction can be found in Douma et al. [17]. Others use the Gaussian linear regression on Beta-values and discuss the *p* values and the false-/true-positive rates [18].

Finally, Xie et al. [19] present different approaches to overcome the problem of biologically non-interpretable estimands as differences in *M*-values $\Delta _{M}$. They propose different algorithms of transforming the $\Delta _{M}$ directly into differences in Beta-values $\Delta _{Beta}$. However, the work lacks a comprehensive comparison of different possible models and a usable implementation.

The aim of the paper is to provide guidance to scientists in the field of DNA methylation analysis. To date, specific guidance for the use of estimands in differential DNA methylation analysis is lacking. The decision to use an estimand may be driven by the bioinformatics analysis pipeline or by the requirement of the research question. We aim to raise awareness of the difficulties that can arise when the two views are not connected. Therefore, we present four “intuitive” approaches and discuss the impact of the choice on the results. Thus, our goal is to facilitate the choice of statistical models and algorithms to integrate statistical significance and biologically informative effect sizes in DNA methylation analysis. Furthermore, we found that the most problematic CpG sites are the hyper- or hypomethylated ones. These sites show DNA methylation levels close to zero and one. This numerical property must be taken into account if the interpretation of the estimates should not become misleading. We illustrate this problem with experimental data and a simulation study. We present the intercept method for a valid transformation of differences in *M*-values into differences in Beta-values [19]. Finally, we demonstrate the problem on two freely available human genome-wide DNA methylation data. The corresponding R code is available on GitHub.

## Results

In the choice of Beta-values or *M*-values for bioinformatical analysis, one must consider two aspects. First, one wants interpretable estimands based on the research question, so that biologically meaningful effect estimates can be reported. Second, one wants statistical packages, which are available to obtain the required estimates from the data to address the research question. In the following, we will therefore look at the problem of the reporting of effect estimates from two different angles: (1) the biologists’ research question and (2) the analytical bioinformatical view using a pipeline of different tools.

We frequently use terms like “beta” in different contexts, which might be confusing for the reader [20]. Therefore, we have defined the used terms and the statistical meaning in Table 3 in the "Methods" section. In addition, a difference between the technology must be made. There are two technologies available: the Illumina DNA methylation assay and bisulfite sequencing. Both types deliver intensities of DNA methylation. The wording differs slightly. The outcome of Illumina DNA methylation assay is called “Beta-values” and the outcome in bisulfite sequencing “methylation levels”: a ratio of methylation on a given CpG site.

### Estimand decision based on research question

Beside the bioinformatics view, the research question should be the main focus of analysis. We focus our work on the unbiased estimand question. Which means that, we do not want to have a sorted *p* value list, but want to obtain a good estimand for each CpG site answering the research question. Typically, the scientist is interested in the effect of some treatment on the DNA methylation at a certain CpG site, i.e., the average difference between two treatment groups per CpG site. The differences in *M*-values do not have any biological meaning. The Beta-values describe the percentage of DNA methylation at a given CpG site. There are now four possible approaches for the generation of meaningful estimands in DNA methylation analysis:

1. Gaussian linear regression on Beta-values,
2. Beta regression on Beta-values,
3. *M*-values for significance, Beta-values for estimands and,
4. Transformation of differences in *M*-values to differences in Beta-values.

To compare these approaches, we performed a simulation with a simple model of a differential DNA methylation analysis, consisting of two treatment levels *placebo* and *verum*. First, we use a model without confounders and then a more complex model including two confounders *age* and *sex*. We run the simulation in a high sample size setting, with each treatment group containing 500 patients. The simulation is described in more detail in the "Methods" section. We also verify the results using experimental data obtained from primary samples.

#### Approach 1: Gaussian linear regression on Beta-values

The approach (1) means simply feeding Beta-values into the standard bioinformatical pipeline. We switch from the asymptotically normal distributed but biologically meaningless *M*-values to the Beta-values. Then, we run the pipeline using minfi (i.e., limma) on Beta-values. Therefore, we generated normal distributed *M*-values and transformed them to Beta-values by Eq. 3. Further information is supplied in the "Methods" section. However, this approach may yield predicted values below 0 or larger than 1, especially when adjustment for continuous variables is performed. Further, since Beta-values are beta-distributed, they tend to show severe heteroscedasticity, violating the assumption of the regression model. On the other hand, linear regression yields estimates for the mean difference in percentage points between groups, which may be an interpretable measure of change in DNA methylation. Depending on the strength of the effect, the *p* values can be significant. However, *p* values should be jointly discussed with an appropriate unbiased effect estimate [6]. Potential effects should be investigated after the initial differential analysis. We repeated the simulation in a mid- and large sample size setting. The overall pattern is the same; the effect estimates from a Gaussian linear regression on Beta-values might be biased, if CpG sites with Beta-values close to 0 and 1 are analyzed. The scientist must verify that the estimands are trustworthy.

#### Approach 2: Beta regression on Beta-values

Approach (2) is calculating a Beta regression on the Beta-values. In this case, the distribution of the Beta-value is taken into account, and the correct regression model is used. This avoids the above-mentioned problems: Beta regression yields predictions in the range of 0 to 1 and has no heteroscedasticity problems. The R package betareg [21] offers a practical implementation. The resulting coefficients must be back-transformed by an inverse logit transformation $\exp (x)/(1+\exp (x))$. The result of the beta regression is then similar to an odds ratio and must be interpreted accordingly, not as a difference in percentage points of DNA methylation, but as the ratio of DNA methylation odds.

Betareg, however, shows severe convergence problems at the borders of the beta distribution. Supplementary figure 1 shows the convergence rates for different $\beta _0$ as mean of the *Placebo* group and an effect to the *Treatments* group of $\beta _1 = 0.1$. Nearly all models will converge, if at least the mean of the *Placebo* group $\beta _0$ is 0.1. Smaller simulated Beta-values tend to result in no-model fit and thus no estimates. If the Beta-values are large enough $>0.1$, the model will produce unbiased effect estimates. Due to symmetry of the Beta distribution, this will be also the case for Beta-values $>0.9$. Hence, the approach (2) is only feasible, if the DNA methylation sites are not mainly hypo- or hypermethylated. Therefore, a filtering step might be a solution in which only CpG sites between a DNA methylation of 0.1 and 0.9 are modeled. Triche et al. [22] show the application of the Beta regression on genome-wide DNA methylation association studies. They showed as a result of enhanced power, and therefore, greater sensitivity to detect changes in DNA methylation can be observed in the simulation studies.

#### Approach 3: *M*-values for significance, Beta-values for estimands

**Fig. 1:**
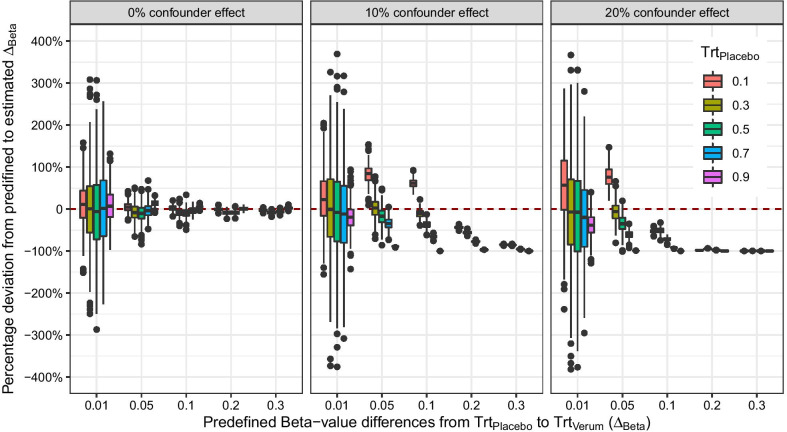
Simulation of the effects estimation influenced by none or two confounder effects. On the y-axis, the percentage deviation from the predefined $\Delta _{Beta}$ to estimated $\hat{\Delta }_{Beta}$ and on the x-axis the raw mean difference of the Beta-values between treatment groups. The first subplot shows the 0% confounder effect. The other two subplot the confounder effects of 10% and 20%. Simulated data with two treatment levels. The deviation is not symmetrical, because the confounder effects were always simulated in the same direction. 5000 simulations with $n=1000$ each

The advice to use the approach (3) is not uncommon [1, 12]. *M*-values are used for calculating *p* values. Beta-values are then used for reporting estimands as differences in Beta-value means. However, reporting raw mean differences not accounting for confounders will result in confounded effect estimates. In the following, we want to answer the question how strong the bias between the estimated effect $\hat{\Delta }_{Beta}$ to the predefined $\Delta _{Beta}$ would be, if we used the mean difference in Beta-values as estimand. Therefore, we run two additional simulation studies (both included in Fig. 1) and check whether we could recover the original effect by simply taking the mean between the two treatment groups. We hypothesize that if a simple model does not deliver satisfying results, the more complex ones (i.e., with a more complex variance structure) will also have problems. Therefore, the most simple model would be a model with one treatment factor and two levels *Placebo* and *Treatments* (Eq. 4), the classical differential analysis setting. Further, we examined a more complex model with two confounders *Age* and *Sex* (Eq. 5).

*Approach 3.1: Differential analysis without confounder* We first run a simple simulation study without any confounder effects. The data consist only of one treatment factor with two levels *Placebo* and *Treatments* (Eq. 4), where each group consists of 500 observations. Further, we let the mean of the placebo group (Grp$_{Placebo}$) run from 0.1 to 0.9 by 0.2 and the effect $\Delta _{Beta}$, i.e., the difference between Grp$_{Placebo}$ and Grp$_{Treatments}$, from 0.005 to 0.3 with different distances. Figure 1 left panel shows the results of the simulation: If no confounders influence the effect of the *Treatments* group, the mean of the raw Beta-values can be used as an estimand for the report of the effects. However, if confounding is present, the simulation shows considerable deviations from the predefined effect.

##### Approach 3.2: Differential analysis with two confounders

The above setting is quite unrealistic. Usually, confounder effects are present. The confounder effects can be caused by different sources like cell composition effect [23, 24]. Further, confounder effects might be chip effects [25] or in general batch effects [26]. A well written overview on confounder adjustment and inference in epidemiology delivers Vanderweele [27]. DNA methylation analysis in particular demands adjustment for batch effects, cell composition, and gender or age effects. These confounding effects might be quite drastic. The more complex model now extends the above model by two confounders, *Age* and *Sex*. We choose *Age* and *Sex* as naming, because both are easy to capture. Both confounders add up to 10% or 20% of the overall effect. Hence, if the confounders have 20% influence, only 80% of the effect is driven by the *Treatments* treatment (Eq. 5). Figure 1 shows the results of the percentual effect confounding by calculating the mean difference of the Beta-values of both treatment levels. If no confounder effect is present, the bias is the same as in Fig. 1. With a combined confounder effect of 10%, the percentage bias will increase to 100% if the predefined mean differences become larger. The effect is more drastic, if the combined confounder effect becomes larger at 20%.

The results in Fig. 1 indicate that the mean Beta-value method is valid only if no confounder effects are present. If the scientist must assume a slight confounder effect, the deviation increases drastically. We cannot recommend using *M*-values for significance and mean differences of “raw” Beta-values for reporting and visualization. A 10% confounder effect will bias the results at high cost of reproducibility.

#### Approach 4: Transformation of differences in *M*-values ($\Delta _M$) to differences in Beta-values ($\Delta _{Beta}$)

Single Beta-values can be transformed into a single *M*-value with a simple formula and vice versa. No bijective dependency, however, exists between differences of *M* values and that of Beta-values. Therefore, coefficients from the *M* values linear regression cannot be directly transformed into Beta-value effects. In fact, any single *M*-value difference can map to a range of Beta-value differences, as visible in Fig. 5.

**Fig. 2:**
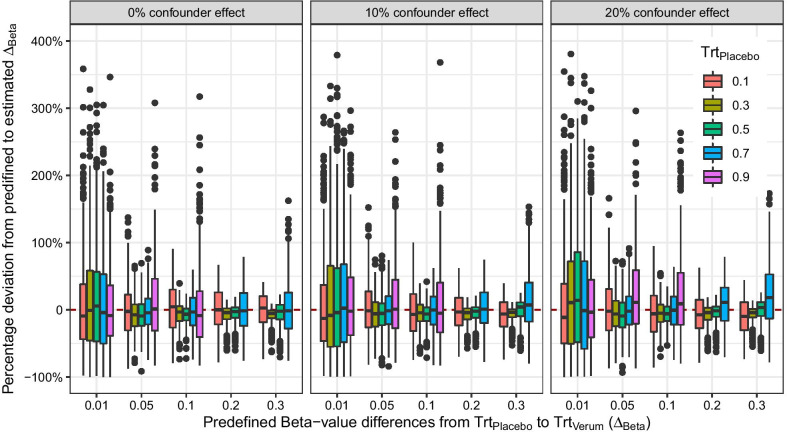
Simulation of the effects estimation with the intercept method and the influence of two confounder effects. On the y-axis the percentage deviation from the predefined $\Delta _{Beta}$ to estimated $\hat{\Delta }_{Beta}$ and on the x-axis the raw mean difference of the Beta-values between treatment groups if we ignoring the confounder effects of 10% and 20%. Simulated data with two treatment levels (5000 simulations with $n=1000$ each)

Xie et al. [19] proposed a different solution by transforming differences in *M*-values to differences in Beta-values. The best results are achieved if the intercept of the regression model is available. Then, differences in *M*-values can easily be transformed to differences in Beta-values by using the intercept of the Gaussian linear regression on *M*-values. In general, the lmFit function of the limma R package omits the intercept from the reporting. However, the functionality is easy to adapt and the intercept can be retrieved with little programming effort. We present R code in supplementary section 3. Figure 2 shows the results of a data simulation with one treatment factor with two levels *Placebo* and *Treatments* with each group consists of 500 observations. In addition, two confounders were added, *Age* and *Sex*. Both confounders add up to 10% or 20% of the overall effect (Eq. 5). If the intercept of the Gaussian regression model is known, the confounder-adjusted *M*-values of the *Placebo* group, i.e., the intercept, can be transformed into Beta-values as well as the *M*-values of the *Treatments* group. Then, the differences between the transformed Beta-values of both treatment levels can be accurately calculated and be reported. We call this approach the intercept method.

### Estimand decision based on bioinformatical analysis pipeline

After the theoretical part and the determination of the method fitting to the research question, the practical part must be solved. The unpracticed scientist might be overwhelmed with the available software solutions in DNA methylation analysis. The consulting bioinformatician might prefer a known pipeline. Therefore, the decision which outcome, Beta-values or *M*-values, to use for a DNA methylation analysis might be determined by the used bioinformatical pipeline and technology. Here, we cannot give a comprehensive overview over all available bioinformatical tools in DNA methylation in respect to estimands, but we try to cover the most popular tools. We therefore refer the reader to Maksimovic et al. [12], who provide a comprehensive overview of a typical DNA methylation analysis workflow and Wreczycka et al. [28], who present strategies for the analysis of bisulfite sequencing data. Heiss et al. [4] discuss the differences between both technologies. Therefore, we will give a broad overview in Table 1 of possible software solutions that might be considered. Table 1 shows a selection of the most frequently used tools in the order of application from the statistical software R and Bioconductor. Different R packages exist for the analysis of DNA methylation and bisulfite sequencing data. Some packages can be used without modification of the raw data; others require a transformation step.

**Table 1:** Overview and guidance on common and selected R packages used in DNA methylation pipelines as a starting point for making decisions based on the desired estimate. See Heiss et al. [4] for information on the differences between Illumina microarrays and bisulfite sequencing. See table 3 for information on *M*-values and Beta-values

R function (Package)	Estimates come from	Used input
**Full pipeline (DMCs and DMRs)**		
BioMethyl$^1$		*M*-values, Beta-values (BS-seq$^{\dagger }$)
minfi$^{2}$		*M*-values, Beta-values
ChAMP$^{3}$		*M*-values, Beta-values
RnBeads$^{4}$		*M*-values (BS-seq$^{\dagger }$)
metilene$^{5}$		Beta-values (BS-seq$^{\dagger }$)
**Preprocessing**$^{\ddagger }$		
ComBat (sva$^6$)		*M*-values (BS-seq$^{\dagger }$)
melon (wateRmelon$^{7}$)		Beta-values
BMIQ (wateRmelon$^{7}$)		Beta-values
SWAN (missMethyl$^{8}$)		Beta-values
CellDMC (EpiDISH$^9$)	lm (stats)	Beta-values
**Detect differentially methylated single CpG sites (DMCs)**		
champ.DMP (ChAMP$^3$)	lmFit (limma$^{10}$)	*M*-values, Beta-values
dmpFinder (minfi$^2$)	lmFit (limma$^{10}$)	*M*-values, Beta-values
calDEG (BioMethyl$^1$)	t-test	*M*-values, Beta-values
varFit (missMethyl$^{8}$)	lmFit (limma$^{10}$)	*M*-values
DMLtest (DSS$^{11}$)		Count values (BS-seq$^{\dagger }$)
**Detect differentially methylated regions (DMRs)**		
bumphunter (bumphunter$^{12}$)	lmFit (limma$^{10}$)	*M*-values(BS-seq$^{\dagger }$, after transformation)
champ.DMR (ChAMP$^3$)	bumphunter (bumphunter$^{12}$)	*M*-values
dmrcate (DMRcate$^{13}$)	lmFit (limma$^5$)	*M*-values (BS-seq$^{\dagger }$)
gometh (missMethyl$^{8}$)		*M*-values, Beta-values
BSmooth (bsseq$^{14}$)		Beta-values (BS-seq$^{\dagger }$)

$^1$Wang [59], $^2$Aryee [32], $^3$Tian [31], $^{4}$Müller [29], $^{5}$Jühling [36],

$^6$Johnson [52], $^{7}$Pidsley [44], $^{8}$Phipson [60], $^9$Zheng [35], $^{10}$Smyth [34],

$^{11}$Park [16] $^{12}$Irizarry [61], $^{13}$Peters [62], $^{14}$Hansen [63]

$^{\dagger }$BS-seq: Supports (processed) bisulfite sequencing data. Packages might need “transformed count data”

$^{\ddagger }$See Assenov [30] for bisulfite sequencing and McEwen [33] for Illumina microarray data

There are different points to consider. The scientist should be aware of the pipeline-like structure of the DNA methylation analysis. Hence, the input of a method is often the output of the immediately preceding method. A standard analysis pipeline starts with preprocessing including quality control and normalization followed by an exploratory analysis including PCA and MDS analysis followed by differential analysis. The results of the differential analyses are then further examined in the genetic context, one example being differentially expressed regions. Therefore, it is important to track what each new method demands: Beta-values or *M*-values. Switching between values and methods might be problematic if the effects, i.e., changes of the values, are of interest. The changing between the values might be not problematic, if *p* value sorted list of CpG sites are the main focus of the analysis.

First, we present complete bioinformatical pipelines with well-established workflows. As an advantage, complete bioinformatical pipelines allow the user to analyze the data in one run. However, if different modules of a given pipeline should be adapted or changed, the user must be aware of the demanded data type. Full pipelines are complex and therefore hard to judge if all the changes between the steps have no effect on the estimand like, for example, the R package RnBeads [29, 30], ChAMP [31], or minfi [32].

Second, we highlight functions from selected and often used R packages. Each module in a complete bioinformatical pipeline can theoretically be changed or offers different options. Especially the preprocessing step has many different approaches available [30, 33]. Often the core method in detection of differentially methylated regions (DMRs) is the well-established R package limma [34]. Therefore, the “main” computations in several R packages are carried out by the function lmFit(). The limma functionality assumes at least asymptotically normal distributed outcome (*Y*) and therefore uses a Gaussian linear regression with variance stabilization. For the estimates to be unbiased, at least asymptotically normal distributed residuals are therefore assumed. Thus, one would naturally use *M*-values as outcome to these functions. Therefore, when the focus is on unbiased estimates, some bioinformatical analysis pipeline require the usage of asymptotically normal distributed *M*-values.

In the following, we highlight some functions and packages. The package EpiDish [35] uses Beta-values as outcome and a Gaussian linear regression for the analysis. The approach should generally be unproblematic if the focus is on generating a variable for confounder adjustment or if very low or very high methylated CpG sites are not considered. Thus, for CpG sites with beta values close to 0 or close to 1. Jühling et al. [36] present a stand-alone package for DMR detection of DNA methylation levels (Beta-values) from bisulfite sequencing data, which uses “absolute DNA methylation ratio (in [0, 1])”. Finally, Park and Wu [16] present the R package DSS, which only accepts count data from bisulfite sequencing and models the data by a negative-binomial or beta-binomial distribution to determine difference in DNA methylation levels, i.e., Beta-values.

We conclude that many but not all bioinformatical tools require an at least asymptotically normally distributed outcome: the *M*-values. In the perspective of estimands, the *M*-values have no biological meaning; however, the intercept method (Approach 4) can be a solution to transform differences in *M*-values to differences in Beta-values (see Supplementary section 3 for the application in R). There are other solution, which model the Beta-values directly. The scientist must weigh which methodology will provide the answers to their research question.

### Example on two real epigenome-wide association studies data sets

The transformation from Beta-values to *M*-values is possible; impossible is the transformation of differences in Beta-values to differences in *M*-values. Table 4 demonstrates the dependencies, and Fig. 5 visualizes them. Now, one might say this is only a theoretical problem. In real data, this problem does not exist. Be it that certain Beta-values always match certain *M*-values or follow a mathematical function. Therefore, we decided to investigate the relationship on two experimental data sets.

So far, we have looked at the problem using simulation data. However, we want to check whether the problem also exists in experimental data. The theoretical *M*-values are real numbers and asymptotically normal distributed; therefore, the differences are also asymptotically normal distributed. In experimental data sets, this might not be the case. It is possible that in real life the distribution of the differences in *M*-values differs from the simulated ones. Therefore, we checked the distribution of *M*-values and possible $\Delta _{M}$’s on two freely available ArrayExpress data sets: the ArrayExpress data set E-GEOD-55763 [37] and the ArrayExpress data set E-GEOD-68379 [38] as a large cancer study, both genome-wide data. Cancer status could have stronger effects on the DNA methylation state, than in normal experiments. E-GEOD-55763 has also a study population and technical replicates. The technical replicates were originally used for data quality issues. Here, we concentrate on the study population.

We used the available processed datasets “GSE68379_Matrix.processed.txt” and “GSE55763_normalized_betas.txt”, which are both preprocessed and should be therefore quality-controlled. Further information on the quality control can be found in the connect references [37, 38]. We provide a R script for the processing on https://github.com/jkruppa/estimands_DNA methylation. The scientist must download the example data from ArrayExpress. E-GEOD-55763 has 2711 samples and 431,961 CpG sites, and E-GEOD-68379 has 1028 samples and 474,517 CpG sites. Table 2 shows the summary of the *M*-values of both data sets. Overall, both data sets seem to have the same distribution, as the summary statistics differ only slightly.

**Table 2:** Summary table of the ArrayExpress data

	Min	1st	Median	Mean	SD	3rd	Max
E-GEOD-55763	$-31.214$	$-3.493$	0.721	$-.327$	3.505	2.598	8.500
E-GEOD-68379	$-15.960$	$-3.334$	0.350	$-0.123$	3.479	2.846	15.974

Further information on the distribution of the Beta-values of both studies can be seen in the corresponding Additional File 1: Sections 4 for E-GEOD-55763 and section 5 for E-GEOD-68379. We present the distribution of the Beta-values for E-GEOD-55763 in supplementary figures 2, 4, and 5 as well as the *M*-values in supplementary figures 3, 6 and 7. Further, the distribution of the Beta-values for study E-GEOD-68379 in supplementary figure 10 and for the *M*-values in figure 11.

**Fig. 3:**
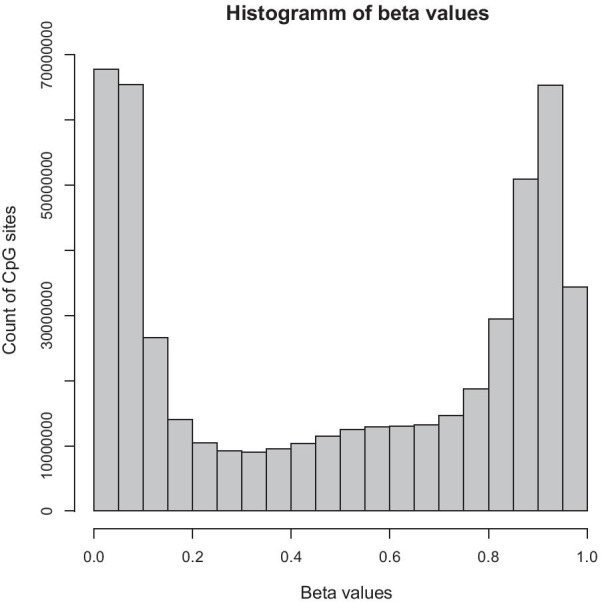
Histogram of the $\beta$-values of the study population of the ArrayExpress data set E-GEOD-68379. This study in particular shows a high number of methylation sites close to 0 and 1, which could be of interest and a problem in modeling

Figure 3 shows an example of the occurrence of hyper- or hypomethylated CpG sites. Therefore, we can observe that there are many CpG sites with Beta-values close to 0 and 1 that are consistent with our simulation results. Additional File 1: Section 5 shows additional figures. The histograms of the M values show the implication of approximately normally distributed. Although there may be a shift, the analyst must conduct the final judgment.

In addition, we wanted to picture the distribution of $\Delta _M$ values from a differential analysis based on a permutation test for both data sets. Therefore, we randomly generated treatment and placebo groups of different sizes each: 5, 10, 20, 50, and 100. We ran a permutation test with 1000 replicates and determined the range of differences in *M*-values. Additional File 1: Figure S8 and S9 show the distribution of $\Delta _{M}$ for the different group sizes of the study population and the technical replicates (*n* = 36) for the ArrayExpress data set E-GEOD-55763. The technical replicates were originally used for data quality issues. Additional File 1: Figure S12 plots the results for the ArrayExpress data set E-GEOD-68379, respectively.

**Fig. 4:**
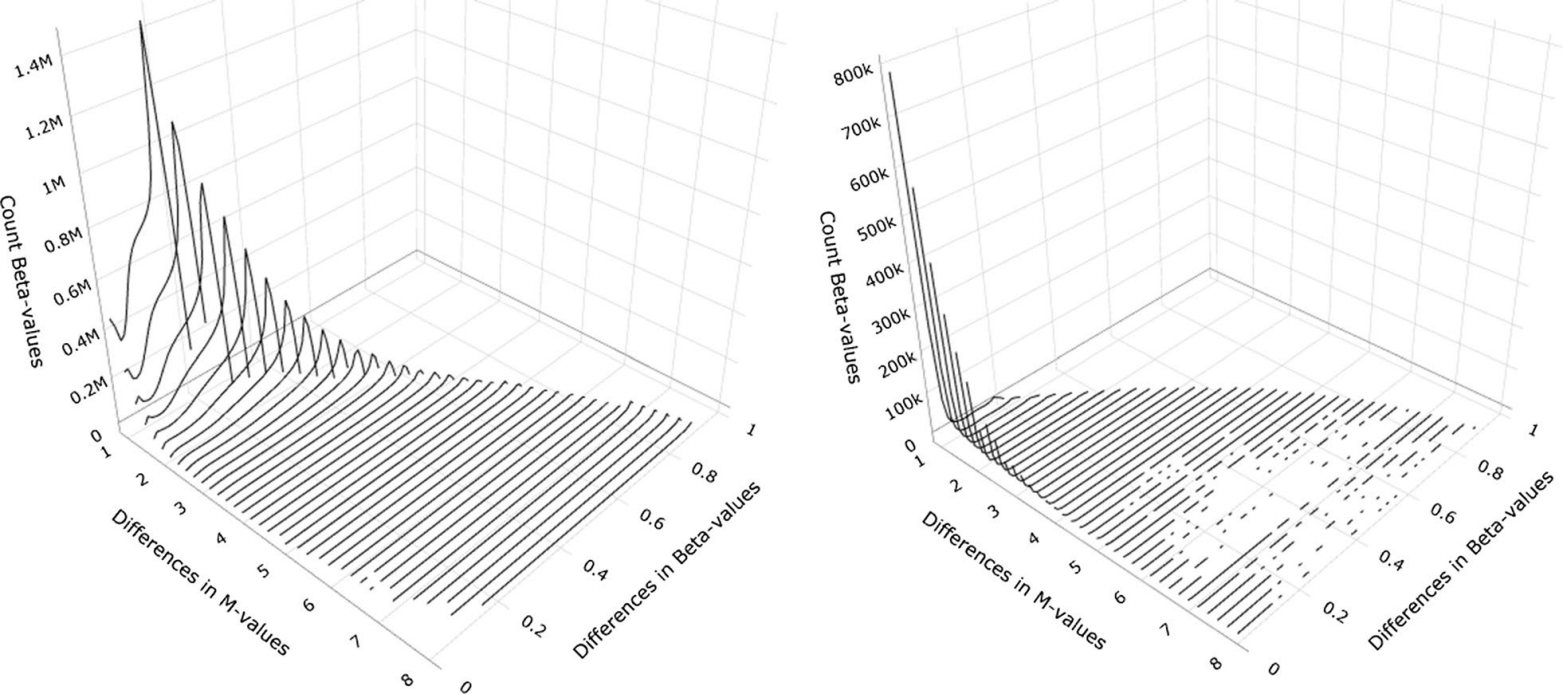
3D surface density plot of the distribution of differences in *M*-values to differences in Beta-values from E-GEOD-55763 (left) and E-GEOD-68379 (right). The difference in *M*-values ($\Delta _{M}$) is mapped to the corresponding differences in Beta-values ($\Delta _{Beta}$) observed in the data set by comparing two groups of five observations each with random group assignment in 5000 simulations . For $\Delta _{M}$ larger than 7, we run 10000 simulations. The small group size of five was chosen for demonstration purposes and is by no means a sufficient group size

We observe the largest effect ranges in differences in *M*-values between both groups in small group sizes of 5. Therefore, we perform differential analyses with a *Treatments* and *Placebo* group size of 5 each. Such a small group size is not realistic, but helps to demonstrate the dependency of *M*-values to Beta-values in experimental data. The small group size of five was chosen for demonstration purposes of effect ranges and is by no means a sufficient group size for future planned EWAS. Therefore, we were able to determine the range of $\Delta _{M}$ generated in both data sets. We see in Fig. 4 the dependency between the differences in *M*-values and corresponding Beta-values for both studies. First, we estimated *M*-values for the *Placebo* and the *Treatments* group. Then, we transformed both *M*-values into the respective Beta-values. This way, we were able to calculate from the known *M*-values the corresponding Beta-values and the differences of the means in both groups. Hence, Fig. 4 shows the 3D plot of the distribution of differences: The $\Delta _{M}$ values are on the x-axis, the corresponding $\Delta _{Beta}$ values on the y-axis, and on the z-axis are counted the number of occurrences for each pair of differences. This is plotted for E-GEOD-55763 (left) and E-GEOD-68379 (right). The differences in *M*-values are mapped to the possible differences in Beta-values observed by the differential analysis. Interestingly, both data sets show different distributions. Therefore, a general pattern cannot be inferred from real-life data sets.

## Discussion

A lot of guidance for DNA methylation analysis has been published. Starting with a global comparison of Beta-values and *M*-values by Du et al. [1], followed by Saadati and Benner [39] with emphasis on Gaussian and Beta regression. Then, Li et al. [40] concentrated on differential DNA methylation analysis with regard to FDR control, power, and stability as well as Mansell et al. [18], which focus on the *p* values using a Gaussian linear regression on Beta-values, and discussed the false-/true-positive rates. All these guidelines have the focus on false discovery rates like *p* values, *q* values, FDR, or power in common. None of them discuss the demands of practical estimands in DNA methylation analysis. Even in practical tutorial-like publications, the issue is not discussed [1, 12]. The reporting of *p* values without estimands, i.e., effect estimates, must be seen critical. *P* values cannot be interpreted in isolation and must be seen in context [5]. In our work, we want to concentrate on the problems of estimands in DNA methylation analysis. However, a comprehensive discussion on effect estimates and *p* values in the context of clinical studies can be found in Pogrow [41].

Choosing the right estimand in a differential DNA methylation analysis is not straightforward. The decision to be made is complex due to two possible usable values and the available bioinformatical software. The inherent assumption of normal distribution is made by the bioinformatical pipeline. In this work, we discussed four approaches of reporting estimands in differential DNA methylation analysis.

The analysis of beta-distributed outcomes with Gaussian linear regression seems counterintuitive. However, the approach has been used in recent research. Salas et al. [42] used Beta-values as outcome and linear mixed models regression as statistical models with confounder adjustment. This is feasible, because the candidate CpG sites have mean Beta-values around 25% and 75% in both groups. Hence, there is a good example of the appropriate usage of a linear regression on Beta-values. Among other criteria, the post hoc enrichment analysis was based on Beta-value differences greater than 0.1 across all cell types. All visualization was done on the Beta-values. A replication of the results seems to have been computed with the same statistical models. In our study, we observed severe convergence problems with low Beta-values when applying the beta regression on Beta-values, resulting in the lack of effect estimates in datasets with smaller differences in DNA methylation patterns. Furthermore, as the estimand of a beta regression is not a difference but a ratio, researchers must be aware of the different interpretation of a difference or a ratio.

Next, for the widely used approach to divide the differential analysis and the reporting of *p* values from the reporting of effect estimates, we showed that the estimands would be biased if confounder effects, a typical feature of biological data, were present. If the sample is large enough, even small clinical irrelevant effects can become significant. We therefore cannot recommend using *M*-values for significance and mean differences of “raw” Beta-values for reporting and visualization. Even a small confounder effect will bias the results at high cost of reproducibility. Finally, the intercept method, based on Xie et al. [19] and refined in this work, allows to calculate the difference in Beta-values from the difference in *M*-values using the intercept estimate of the limma model, providing estimands that take the effects of confounders into account. The transformation is easy to accomplish, but not available in common software packages. We provide a easy-to-use R code example using the limma package in Additional File 1.

Bisulfite sequencing datasets are based on alignment of reads to a reference genome. Therefore, the determination of the degree of DNA methylation is technically different. However, the final measure is a percentage of DNA methylation at a given position, i.e., CpG site. Therefore, the result of bisulfite sequencing can also be converted to *M*-values. The here presented and discussed numeric properties would be the same; however, the data processing is largely different in bisulfite sequencing and may affect the differential analysis. It is important to note that there are differences to probe design of the illumina arrays, which is important to account for [43, 44]. Zhou et al. [45] provide an overview of a DNA methylation pipeline with bisulfite sequencing. Interestingly, bisulfite sequencing data are often called DNA methylation levels or proportion, which can be named Beta-values. The different naming makes sense, because of the different context of read counts and signal intensities.

In addition to discussing the proper estimation of effects in clinical trials, we also discuss the influence of normalization methods on final results. So, do we model the noise caused by preprocessing (e.g., normalization and filtering) or the biological effect? Or is the noise effect more important than the choice of statistical model? Hancock et al. [46] discussed the issue in a broader sense and Qin et al. [47] with emphasizes to omics data. In particular, for DNA methylation analyses, the confounder effect of cell composition must be considered [48, 49]. Other confounding factors that should be considered are batch effects [50]. Depending on the study type and the patient collective study, specific confounders might be needed. Finally, Mishra et al. [51] discuss the global goals of data preprocessing. The work of Mishra et al. (2020) is in the context of chemometric models, but provides a comprehensive overview of the general selection process strategies of preprocessing methods. We decided to use confounder effects which make effects. Finally, we chose confounder effects that clearly led to differences in the simulation study. These effects may be too high or too low. However, this evaluation of confounder effects also depends on the experiment conducted, design, and tissue used. Researchers should know the effect of confounders on the effect estimators and consider them in the interpretation [7, 35, 52].

If the research question is based on a “*p* value” ranked CpG site list, we recommend the work of Van Rooij et al. [7] as a complement to our work. For error rates, Van Rooij et al. [7] evaluated different statistical models and methylation values as well as the effects of confounding. In addition, they discuss the results in the context of RNAseq. Van Rooij et al. [7] found that no methylation value transformation has a large impact on the ranking by error rates. They recommend beta-3IQR values, i.e., Beta-values without extreme values. Van Rooij et al. [7] do not discuss effect estimates because they are not within the scope of their work.

In terms of the research question, the researcher could focus on specific CpG sites. It might be possible to focus on CpG sites with Beta-values close to 0 and 1 and dichotomize the CpG sites into a binary indicator. After dichotomization, a Fisher exact test would be possible. Again, the estimate of an exact Fisher test is an odds ratio and the definition of the binary indicator must meet the requirements of the scientist. This approach may be of interest as our analysis of E-GEOD-68379 may serve as an example.

We cannot cover all issues connected with biased reported estimands. We consider the combination of different clinical studies in a meta analysis as one of them. Therefore, the highest value of evidence can be reached with meta analysis and systematic reviews. If a meta analysis should be run, two settings must be distinguished: (1) all data of the studies are available and can be reanalyzed or (2) only the publication is available and effect estimates should be combined. It is very important to distinguish between DNA methylation measurements as outcome [53, 54] or as risk factor [55]. In our work, we concentrate on DNA methylation measurements as outcome. While single studies might have a lack of reproducibility, the combination of different single studies can be an impossible challenge due to differences in processing pipelines and statistical models. As an example, Morris et al. [56] discuss the epigenetic landscape of renal cancer. There are no estimands reported for the DNA methylation part. Instead, more a general scheme of up- and down regulation by CpG islands connected to promotor regions. Kerr et al. [57] stated, in their recent review on rare renal diseases, that the methodical rigor was weak in all thirteen considered studies. The information on the DNA methylation measurement method is reported for each study, but this does not help to judge the estimands in each study as a lack of accounting for confounding factors can be found in all case–control studies even if the factors are mentioned. They conclude that “future studies would benefit from standardization of the detection and analysis of methylation, [...] and a comprehensive, transparent reporting structure”. A template might be the STREGA statement, which provides the scientific community with a checklist for the performance of genome-wide association studies to enhance the transparency of its reporting, regardless of choices made during design, conduct, or analysis [58]. With this work, we aim to facilitate the choice of correct estimands for specific DNA methylation analyses and therefore add to more standardized analysis workflows, enhancing comparability and reproducibility across different studies.

## Conclusion

Many bioinformatical DNA methylation analysis pipelines demand the usage of an asymptotically normal distributed outcome. The outcome should be asymptotically normal because commonly used R packages are based on the R package limma and therefore have the inherent assumption of normally distributed outcome. So far, methodically benchmarks are done on false discovery rates, which might not be affected by the use of Beta-values analyzed by Gaussian linear regression analysis. This might be the reason of a low number of CpG sites close to 0 and 1 or the usage of robust methods. Nevertheless, the question remains, if the estimands are unbaised. However, we show that confounder effects will bias the effect estimates. In addition, the usage of the technology might also influence the choice of the appropriate estimand. With our study, we come to the following recommendations. *M*-values should be used if significance is a filter for post hoc analysis like pathway analysis or the detection of interesting CpG sites. In a next step, we recommend the usage of Beta-values in a Beta regression to estimate the effects of the CpG sites scrutinized, where the estimand has to be interpreted as an odds ratio. In this context, it has to be emphasized that the Beta regression has problems of modeling values at the borders of the 0 and 1 distribution, i.e., if a CpG site has mostly high methylation or no methylation. In this case, estimating the effect in terms of differences in Beta-values may also be achieved by using the intercept method.

Therefore, depending on the experimental setting and the connected research question, *M*-values or Beta-values can be used as outcome. In no case should *M*-values be used for determination of the significance and raw Beta-value differences as effect measure. The estimands on Beta-values will be biased, if even a small confounder effect is present. We want to encourage scientist to choose the estimand, which fits best to the research question and the biological model. We see similar mathematical symbols and statistical word usage in DNA methylation for different concepts, which can lead to unnecessary confusion. With our work, we hope to facilitate the collaboration and planning of further clinical trials.

## Methods

### Statistical wording in DNA methylation analysis

Some statistical wording in DNA methylation analysis is special, because one of the measured outcomes is called “beta”. Therefore, in our article we will frequently use statistical terms like “beta” in a different context which might be confusing for the reader [20]. Therefore, we have defined the used terms and the statistical meaning in Table 3. A DNA methylation analysis can consist of hundreds of thousands of CpG sites. Each i$^{th}$ CpG site has a single Beta$_i$ value. Each of the single Beta-values can be transformed into *M*-values. In general, the Beta- and *M*-values are the outcome of the DNA methylation analysis. In our article, we concentrate on the differences between *M*-values and Beta-values defined as $\Delta _M$ and $\Delta _{Beta}$, respectively. We call these differences in Beta- and *M*-values estimands, because the differences are “what is to be estimated” [9]. Further, a linear regression will produce estimates for the intercept $\beta _0$ and the effect estimate $\beta _1$ for the treatment effect, i.e., the difference between the *Placebo* and *Treatment*.

**Table 3:** Table of used terms, their statistical meaning, and description

Term	Description and usage
Beta-values	Describe the frequency of methylation at a given CpG site. Numeric values between 0 and 1. Biological interpretable.
Beta$_i$	Single Beta-value *i* of all *p* Beta-values
*M*-values	Standardized Beta-values. The standardization must be read as “logit” transformation. Numeric values from $-\infty$ to $+\infty$. No biological meaning.
M$_i$	Single M-value *i* of all *p* *M*-values
Outcome	Dependent variable *Y* of the regression models; here Beta-values or *M*-values
$\Delta _{Beta}$	Difference in Beta-values
$\Delta _{M}$	Difference in *M*-values
$\beta _0$, $\beta _1$	Coefficients of the regression model; $\beta _0$ as the intercept and $\beta _1$ as the effect estimate, i.e., the mean difference between the two groups *Placebo* and *Treatment*.

### Transformation of *M*-values and Beta-values

In the following, we briefly describe Beta-values, *M*-values and the differences in them as estimands, respectively. We recommend as introduction to the topic of Beta- and *M*-values the work of Du et al. [1]. Maksimovic et al. [12] can be recommended for a deeper discussion of potential bioinformatical analysis pipeline.

In an analysis of Illumina Infinium DNA methylation arrays *methylated* and *unmethylated* intensities are produced. The fraction of methylated to unmethylated probes for a given CpG site is defined by the Beta-values. The Beta-values describe the percentage of DNA methylation for a given CpG site. The Beta-values can be calculated as follows.

1 $$\begin{aligned} \hbox {Beta}_i = \frac{\max (methylated, 0)}{\max (methylated, 0) + \max (unmethylated, 0) + 100} \end{aligned}$$

The Beta-values are a probability and therefore limited to a range of 0 to 1. Consequently, they are Beta-distributed. The Beta-values can be standardized to *M*-values as follows.

2 $$\begin{aligned} \hbox {M}_i = \log _2\left( \frac{\hbox {Beta}_i}{1 - \hbox {Beta}_i}\right) \end{aligned}$$

The *M*-values are asymptotically normal distributed after the $\log _2$-transformation. The *M*-values can be back-transformed.

3 $$\begin{aligned} \hbox {Beta}_i = \frac{2^{\hbox {M}_i}}{1 + 2^{\hbox {M}_i}} \end{aligned}$$

**Fig. 5:**
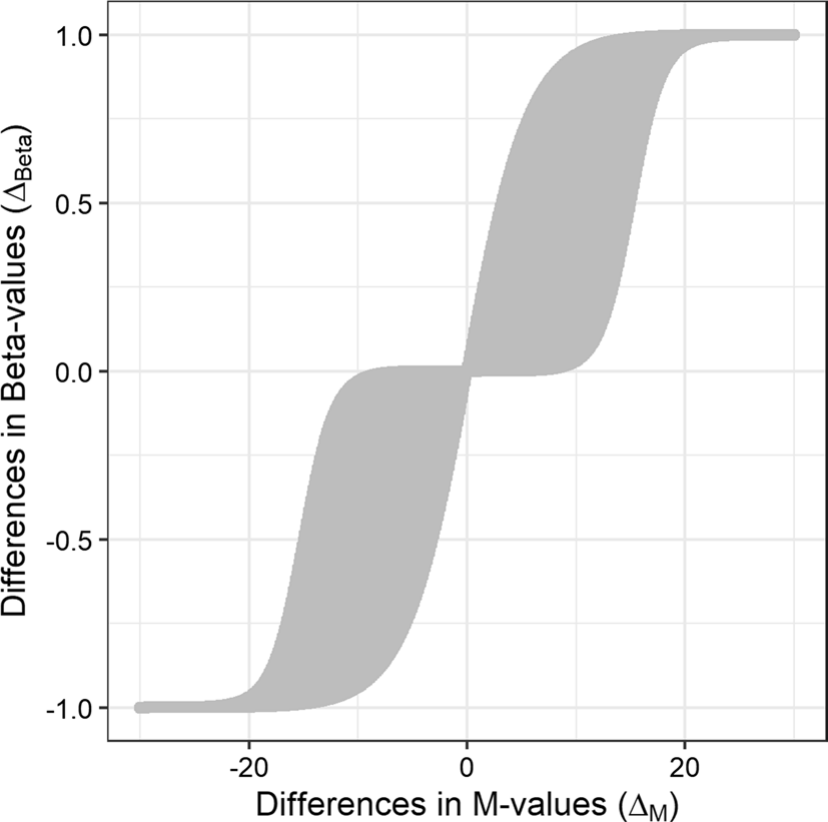
Mustache plot of the theoretical relation of differences in *M*-values to differences in Beta-values. On the left side, the difference in *M*-values ($\Delta _{M}$) is mapped to all possible corresponding differences in Beta-values ($\Delta _{Beta}$). A difference of $\Delta _{M} = 5$, for example, can be mapped to a $\Delta _{Beta}$ from 0.0009 to 0.6996

Counterintuitively, the differences in Beta-values cannot be transformed into differences in *M*-values and vice versa. First, we have examined the theoretical distribution of the $\Delta _{M}$ of a linear regression analysis to the corresponding possible $\Delta _{Beta}$. We demonstrate in Fig. 5 the mustache-like plot of the theoretical distribution. A direct translation of $\Delta _{M}$ to $\Delta _{Beta}$ is not possible. The difference of $\Delta _{M} = 5$ can be represented by a $\Delta _{Beta}$ from 0.0009 to 0.6996. Due to the fact that the mustache plot is symmetrical we will concentrate on the positive differences of the $\Delta _{M}$ values.

Table 4 shows the numeric dependencies between the Beta- and *M*-values as well as the differences. The $\Delta _{Beta}$ is always 0.1 between the *Placebo* and *Treatment* group of the treatment. The $\Delta _{M}$ value depends on the Beta-value of the *Placebo* and the *Treatment*. Therefore, single $\Delta _{M}$ values cannot be matched to single $\Delta _{Beta}$ values. In the last column, the respective regression model on *M*-values is shown. As Xie et al. [19] are pointing out, the best way to achieve the differences of Beta-values out of a Gaussian linear regression on *M*-values is to back transform the estimates of the regression. As an example, the regression formula on *M*-values with $-3.15 + 1.16 \cdot Grp_{Treatment}$ has a $\beta _0 = -3.15$, the mean of the *Placebo* group, and $\beta _1 = 1.16$ the difference between the mean of the *Placebo* and *Treatment* group. Hence, the mean of the *Treatment* group would be $-3.15 + 1.16 = -1.99$ as shown in the table. Now, the mean of the *Placebo* group of $-3.15$ can be back-transformed to 0.101 and the mean of the *Treatment* group of $-1.99$ to 0.201, respectively. Then, it is possible to calculate the differences in Beta-values of 0.1.

**Table 4:** Table of example for the transformation of Beta-Values to *M*-values and the differences, respectively. The Beta-value difference between the Placebo group and the Treatments group is constant at 10%. Due to the transformation, the *M*-values differ and the differences in *M*-values can not be mapped to the differences in Beta-values

Grp$_{Placebo}$		Grp$_{Treatment}$		$\Delta _{Beta}$	$\Delta _{M}$	Regression formula
Beta-value	M-value	Beta-value	M-value			on *M*-values
0.001	$-$9.96	0.101	$-$3.15	0.10	6.81	$-$9.96 + 6.81 $\cdot$ Grp$_{Treatment}$
0.101	$-$3.15	0.201	$-$1.99	0.10	1.16	$-$3.15 + 1.16 $\cdot$ Grp$_{Treatment}$
0.201	$-$1.99	0.301	$-$1.22	0.10	0.77	$-$1.99 + 0.77 $\cdot$ Grp$_{Treatment}$
0.301	$-$1.22	0.401	$-$0.58	0.10	0.64	$-$1.22 + 0.64 $\cdot$ Grp$_{Treatment}$
0.401	$-$0.58	0.501	0.01	0.10	0.59	$-$0.58 + 0.59 $\cdot$ Grp$_{Treatment}$
0.501	0.01	0.601	0.59	0.10	0.58	0.01 + 0.58 $\cdot$ Grp$_{Treatment}$
0.601	0.59	0.701	1.23	0.10	0.64	0.59 + 0.64 $\cdot$ Grp$_{Treatment}$
0.701	1.23	0.801	2.01	0.10	0.78	1.23 + 0.78 $\cdot$ Grp$_{Treatment}$
0.801	2.01	0.901	3.19	0.10	1.18	2.01 + 1.18 $\cdot$ Grp$_{Treatment}$
0.901	3.19	0.999	9.96	0.10	6.77	3.19 + 6.77 $\cdot$ Grp$_{Treatment}$

### Used simulation models

In the following, we describe the simulation approach mathematically, for those who have a better access via programming code the simulation R code is available on https://github.com/jkruppa/estimands_methylation. We used two different simulation models for the comparison of the predefined and estimated effects. First, a simple model on which we can discuss the advantages and disadvantages of the approach. The results can be seen in Fig. 1 on the left panel.

4 $$\begin{aligned} Outcome = \beta _0 + \beta _1 \cdot Grp + \epsilon \end{aligned}$$

where

- Outcome represents the measured *M*-values or Beta-values for one CpG site
- $\beta _0$ is the intercept of the regression model and the mean of the *Placebo* group
- $\beta _1$ is the effect estimate, i.e., estimand, of the *Treatment* group representing the mean difference between *Placebo* and *Treatment*.

The regression model (4) is very simple and can also be seen as a *t*-test. However, standard bioinformatical pipelines often use the Gaussian linear regression with variance correction for the differential analysis [34]. The reason is that regression models can be adjusted for confounders like age and sex. The confounder effects are normally not of interest and are discarded.

5 $$\begin{aligned} \hbox {Outcome} = \beta _0 + \beta _1 \cdot Grp + \beta _3 \cdot Age + \beta _4 \cdot Sex + \epsilon \end{aligned}$$

where

- Outcome represents the measured *M*-values or Beta-values for one CpG site
- $\beta _0$ is the intercept of the regression model and the mean of the *Placebo* group
- $\beta _1$ is the effect estimate, i.e., estimand, of the *Treatment* group representing the mean difference between *Placebo* and *Treatment* ($\Delta _{M}$)
- $\beta _3$ and $\beta _4$ are the effect estimates of the confounder, i.e., *Age* and *Sex*.

The approaches (1) to (4) are tested on both models, and the implications were discussed. The overall data generating was done in the environment of the R package simstudy (https://kgoldfeld.github.io/simstudy/index.html). We used the simstudy setup for the data generation. First, the Outcome has been generated as normally distributed (dist = normal). If Beta-values were needed, the normally distributed *M*-values were transformed to Beta-values using Eq. 3. In the case of the analysis of the convergence rate of the Beta regression, betareg(), we generated a Beta distributed Outcome (dist = beta).

Table 4 shows the data generation setting for an effect $\Delta _{Beta}$ of 0.1 between Grp$_{Placebo}$ and Grp$_{Treatment}$. In the next step, we generated the Beta-values for the placebo group and added the effect to achieve the Beta-value for the case group. The difference is always 0.1 as the predefined effect. We use the *M*-values to generate the regression formula and the normal distributed outcome as pictured in Table 4. The regression formula represents the difference of Beta-values of 0.1 in the space of the *M*-values. We are then able to back transform the Outcome to Beta-values and use them as outcome. The effect $\Delta _{Beta}$ is varied in the simulation study. Further, we generated a confounder effect matrix Eq. 6. The confounder effects are positive defined. Therefore, if we ignore the confounder effects, our estimates should have a negative deviation, which can be seen in Fig. 1.6

Depending on the confounder effect, the treatment effect is reduced by the portion shown in Eq. 6. We decided to use a categorical and continuous variable as possible confounders. Figure 1 shows the simulation results of the different confounder effects.

## Supplementary information

**Additional File 1.** Supplementary material including R code and additional figures.

## Data Availability

Online as supplementary material. The example data can be accessed on ArrayExpress by the identifier. Code chunks and further information are also available from https://github.com/jkruppa/estimands_methylation

## Abbreviations

BS-seq: bisulfite sequencing
CpG site: position of a methylation
DMCs: differentially methylated cytosine sites
DMRs: differentially methylated regions
DNA: deoxyribonucleic acid
EWAS: epigenome-wide association study
MDS: multidimensional scaling
PCA: principal component analysis
