# Supplementary material for: Estimands in epigenome-wide association studies
Source: Clin Epigenetics. 2021 Apr 29;13:98. doi: 10.1186/s13148-021-01083-9 (PMC8086103; doi:10.1186/s13148-021-01083-9)
Supplement: Supplementary file 1 — Additional File 1. Supplementary material including R code and additional figures. [file 13148_2021_1083_MOESM1_ESM.pdf]

Supplementary material

Estimands in epigenome-wide association studies

Jochen Kruppa[1,2*], Miriam Sieg[1,2], Gesa Richter[3], and Anne Pohrt[1,2]

[1]Charité - Universitätsmedizin Berlin, corporate member of Freie Universität Berlin, Humboldt-Universität zu Berlin, and Berlin Institute of Health, Institute of Biometry and Clinical Epidemiology, Berlin, Germany
[2]Berlin Institute of Health (BIH), Berlin, Germany
[3]Department of Periodontology and Synoptic Dentistry, Institute of Dental, Oral and Maxillary Medicine, Charité - University Medicine
[*]corresponding author

Contents

1 Overview of the supplementary material 3

2 Additional figures to Beta linear regression 4

3 Intercept method in R - Limma adjustment 5

4 Additional figures to E-GEOD-55763 6

5 Additional figures to E-GEOD-68379 9

1 Overview of the supplementary material

In the following additional material of the paper is provided. We show in additional figures to E-GEOD-55763 and additional figures to E-GEOD-68379.

Supplementary tables No delivered

Supplementary figures Figure 1 and 2 showing the distribution of the β-values and m-values for the full population of the ArrayExpress data set E-GEOD-55763. Figure 3 and figure 4 show the distribution of the β-values for the study population and the technical replication. Figure 5 and figure 6 pictures the distribution of the m-values for the study population and the technical replication. Figure 7 and figure 8 show the maximal and minimal group differences in m-values with group sizes $n_{group} \in \{5, 10, 20, 50, 100\}$ from a permutation test with 1000 replicates of the ArrayExpress data set E-GEOD-55763 in the study population. Figure 8 in the technical replication, respectively.

Figure 9 and 10 showing the distribution of the β-values and m-values of the ArrayExpress data set E-GEOD-68379. Figure 11 show the maximal and minimal group differences in m-values with group sizes $n_{group} \in \{5, 10, 20, 50, 100\}$ from a permutation test with 1000 replicates of the ArrayExpress data set E-GEOD-68379 in the study population.

Figure 12 shows the bias and the convergence rates of a Beta linear regression on the borders of the Beta distribution.

2 Additional figures to Beta linear regression

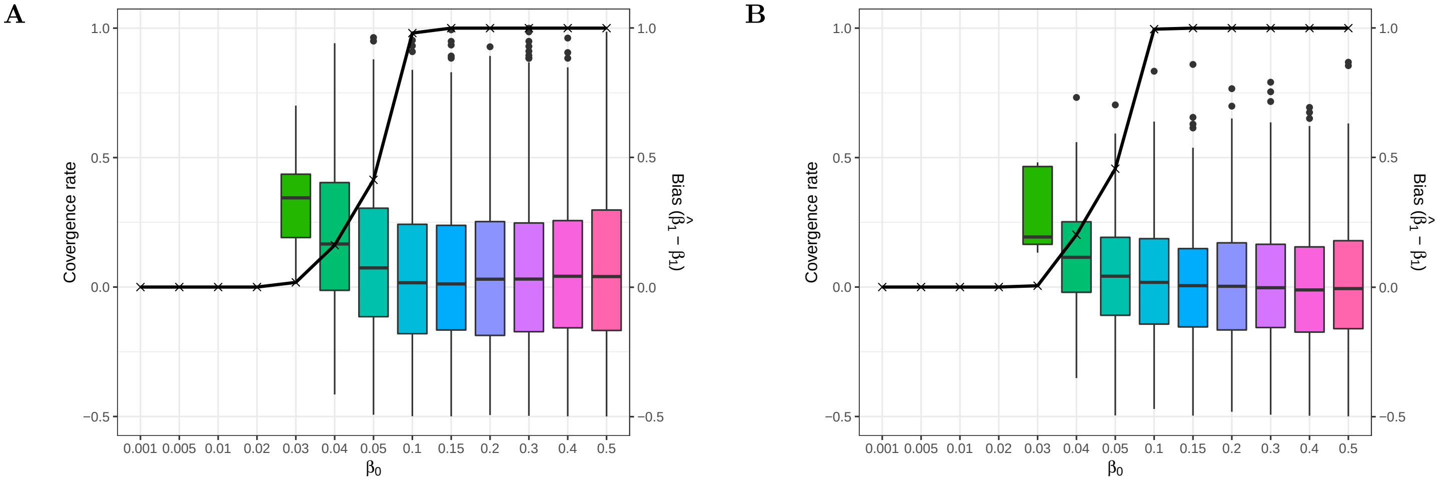

Supplementary Figure 1: Simulation of Beta linear regression with different β_0 and a constant treatment effect $\beta_1 = 0.1$. On the left side, the convergence rate of the model is shown represented by the solid black line. On the right side, the bias is shown as difference between the predefined β_1 and the estimated one $\hat{\beta}_1$ represented by boxplots. The sample size on the subplot 1.A was 50 per treatment group and one the subplot 1.B the sample size was 100 per treatment

3 Intercept method in R - Limma adjustment

For more information see also Du et al. (2008)[1] for M-values and Beta-values in general and Xie et al. (2018) [2] for a theoretical discussion of the intercept method.

```
m2beta <- function (m) {
    beta <- 2^m/(2^m + 1)
    return(beta)
}

beta2m <- function(beta) {
    m <- log2(beta/(1 - beta))
    return(m)
}


exprContr <- matrix(rnorm(100, 5, 2), ncol = 10)
exprCase <- matrix(rnorm(100, 10, 2), ncol = 10)
exprMat <- cbind(exprContr, exprCase)

phenoDf <- data.frame(trt = rep(c("contr", "case"), each = 10),
                      gender = sample(c("w", "m"), 20, replace = TRUE),
                      age = rnorm(20, 50, 10))

limmaDesign <- with(phenoDf, model.matrix(~ trt + age + gender))

limmaFit <- lmFit(exprMat, limmaDesign)

eBayes(limmaFit)

topTable(eBayes(limmaFit))

## use the intercept method to transform the confounder adjusted M-values to Beta-values
lim_fit_coef <- eBayes(limmaFit)$coefficients
m0_eff <- lim_fit_coef[, "(Intercept)"]
m1_eff <- m0_eff + lim_fit_coef[, "trtcontr"]
mean_beta <- abs(m2beta(m0_eff) - m2beta(m1_eff))

R> mean_beta
 [1] 0.6671400901 0.1545557865 0.0641230664 0.0030063165 0.0129515048
 [6] 0.0222119804 0.0903598241 0.0290386186 0.0201646441 0.0427456773
```

4 Additional figures to E-GEOD-55763

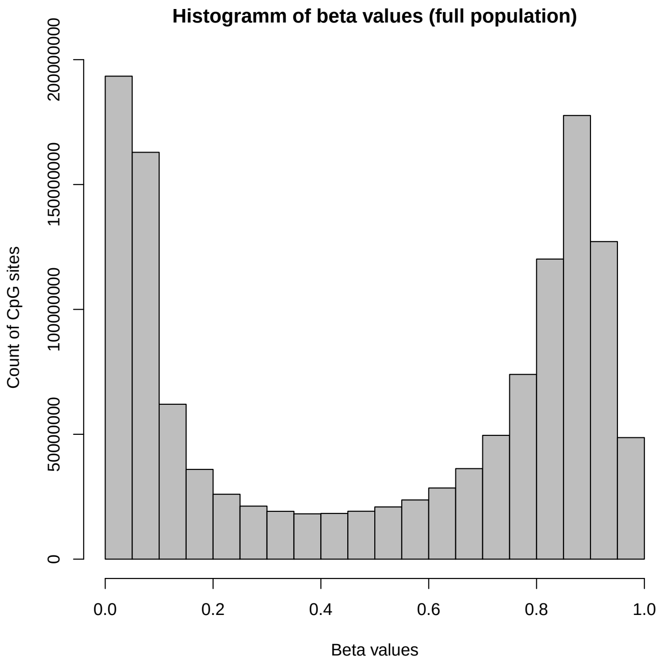

Supplementary Figure 2: Histogram of the β-values of the full population of the ArrayExpress data set E-GEOD-55763.

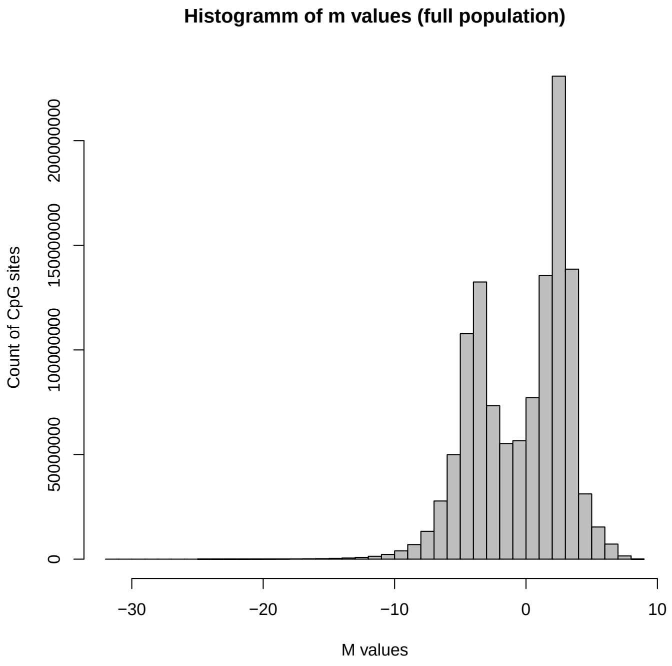

Supplementary Figure 3: Histogram of the m-values of the full population of the ArrayExpress data set E-GEOD-55763.

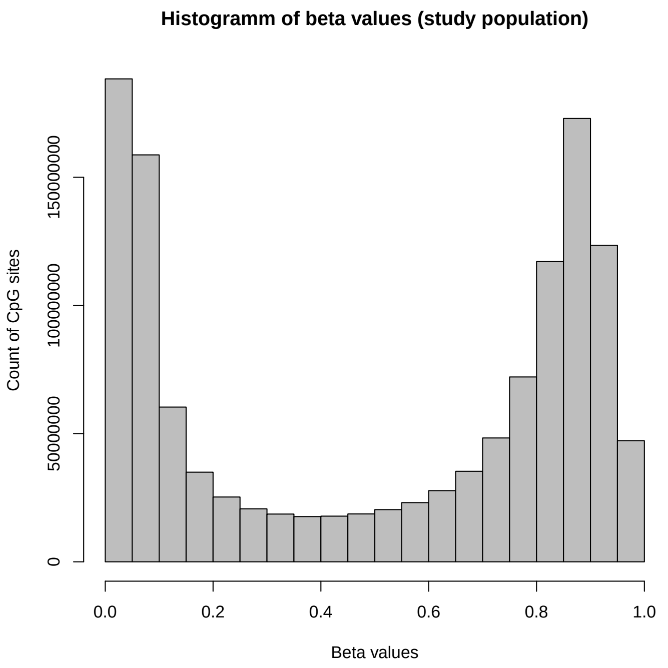

Supplementary Figure 4: Histogram of the β-values of the study population of the ArrayExpress data set E-GEOD-55763.

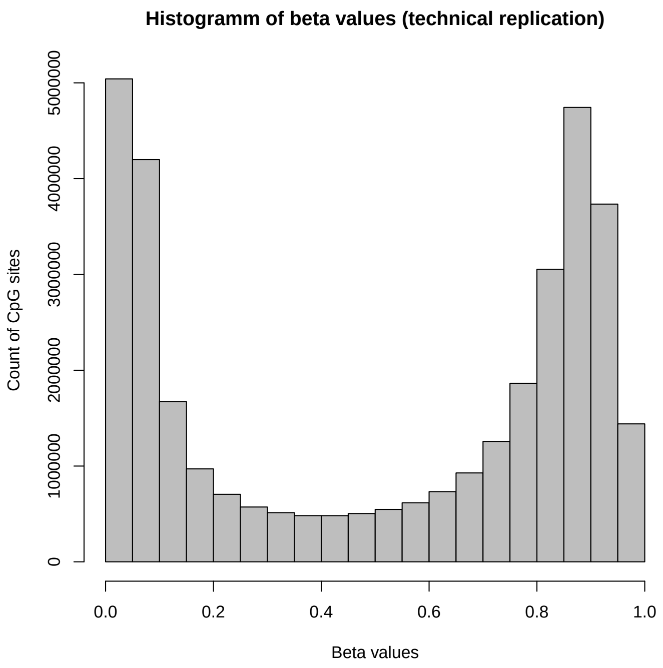

Supplementary Figure 5: Histogram of the β-values of the technical replication of the ArrayExpress data set E-GEOD-55763.

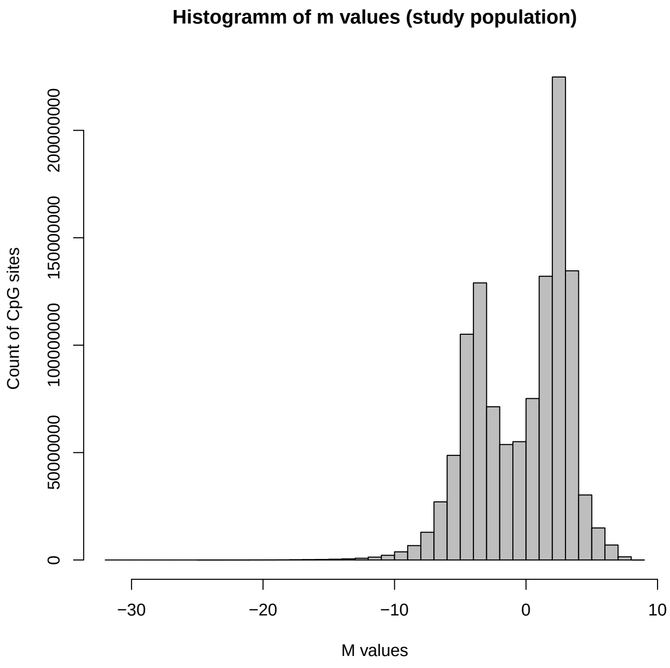

Supplementary Figure 6: Histogram of the m-values of the study population of the ArrayExpress data set E-GEOD-55763.

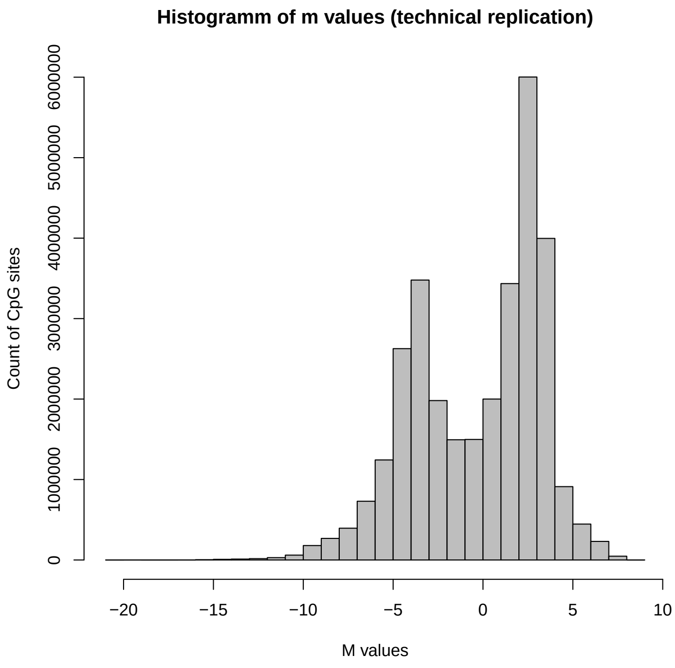

Supplementary Figure 7: Histogram of the m-values of the technical replication of the ArrayExpress data set E-GEOD-55763.

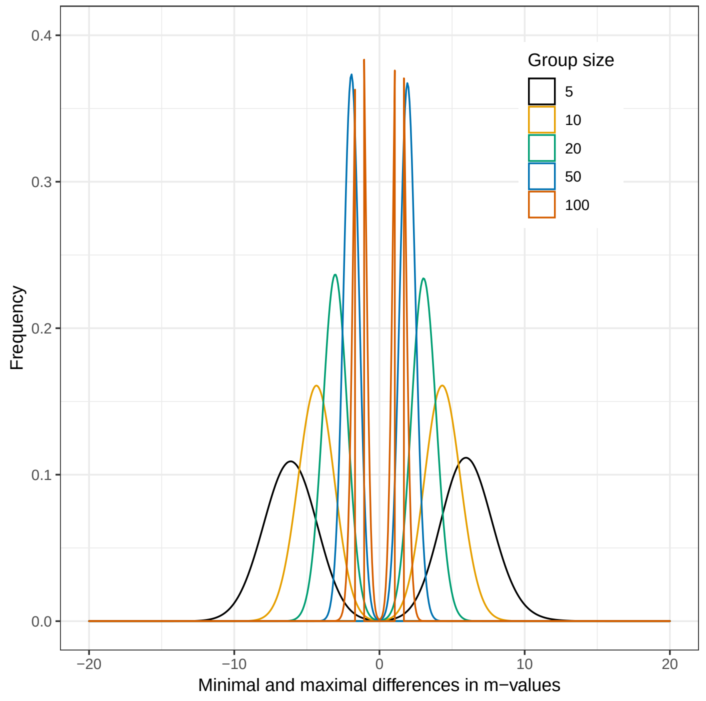

Supplementary Figure 8: Maximal and minimal group differences in m-values with group sizes $n_{group} \in \{5, 10, 20, 50, 100\}$ from a permutation test with 1000 replicates of the ArrayExpress data set E-GEOD-55763 in the study population.

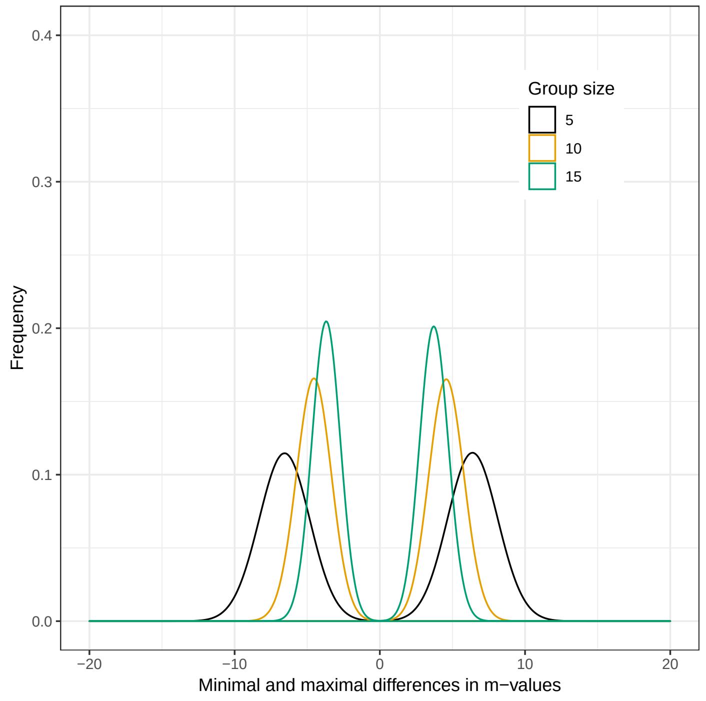

Supplementary Figure 9: Maximal and minimal group differences in m-values with group sizes $n_{group} \in \{5, 10, 20, 50, 100\}$ from a permutation test with 1000 replicates of the ArrayExpress data set E-GEOD-55763 in the technical replicates.

5 Additional figures to E-GEOD-68379

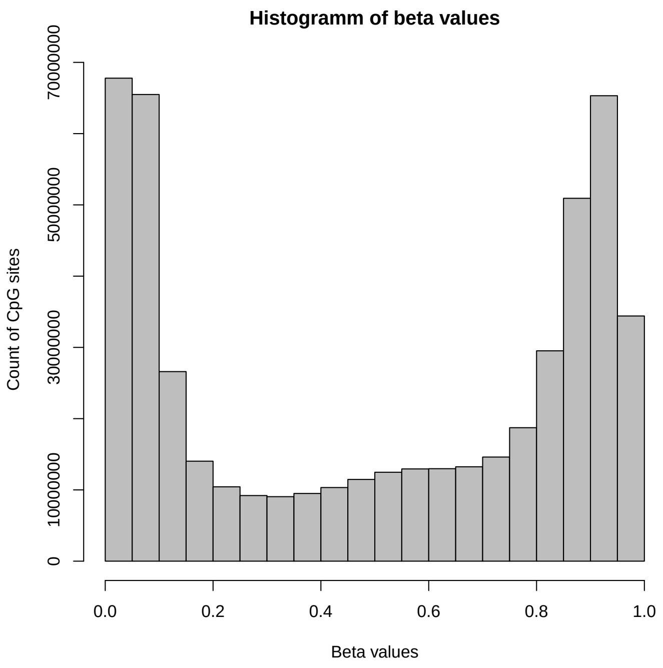

Supplementary Figure 10: Histogram of the β-values of the study population of the ArrayExpress data set E-GEOD-68379.

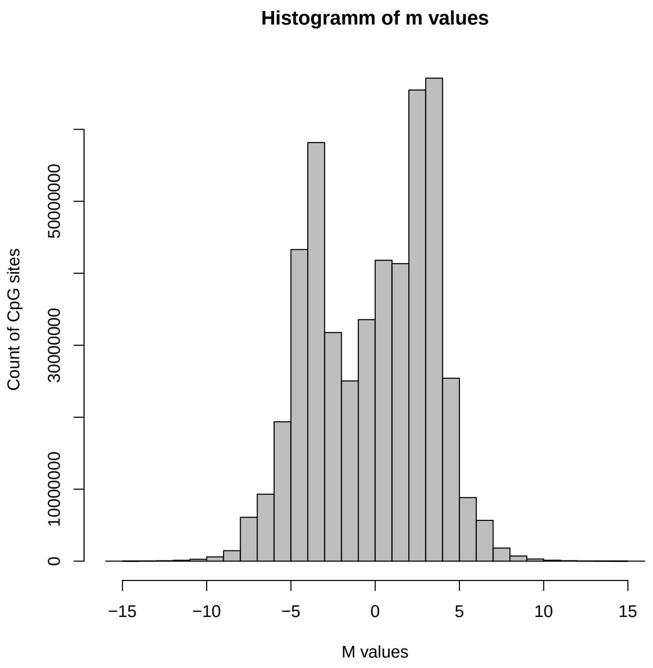

Supplementary Figure 11: Histogram of the m-values of the study population of the ArrayExpress data set E-GEOD-68379.

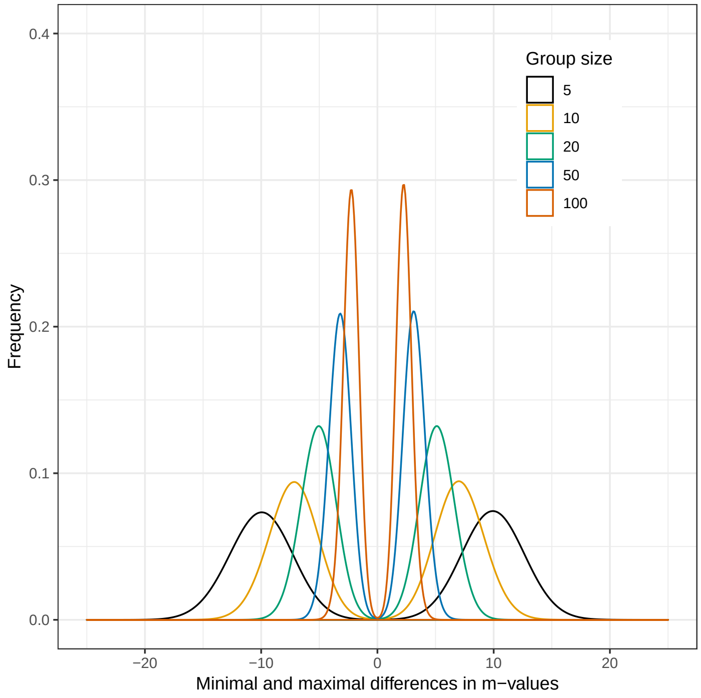

Supplementary Figure 12: Maximal and minimal group differences in m-values with group sizes $n_{group} \in \{5, 10, 20, 50, 100\}$ from a permutation test with 1000 replicates of the ArrayExpress data set E-GEOD-68379.

References

[1] Pan Du, Warren A Kibbe, and Simon M Lin. lumi: a pipeline for processing illumina microarray. *Bioinformatics*, 24(13):1547–1548, 2008.

[2] Changchun Xie, Yuet-Kin Leung, Aimin Chen, Ding-Xin Long, Catherine Hoyo, and Shuk-Mei Ho. Differential methylation values in differential methylation analysis. *Bioinformatics*, 35(7):1094–1097, 2018.
